# Supplementary material for: Are carbon emissions trading and green financial instruments synergistic? -Comprehensive quantitative research based on content analysis
Source: PLoS One. 2024 Mar 7;19(3):e0298601. doi: 10.1371/journal.pone.0298601 (PMC10919601; doi:10.1371/journal.pone.0298601)
Supplement: S1 Data — (ZIP) [file pone.0298601.s001.zip › Data/地级市Z_F工作报告.zip!/╝¬┴╓/░╫│╟╩╨/└┤╫╘╣1⁄2╓┌║┼í╛┬φ┐╦╩2╛▌═°í┐_2011.docx]

各位代表：

　　现在，我代表白城市人民政府，向大会作政府工作报告，请予审议，并请各位政协委员提出意见。

　　一、2010年工作回顾

　　2010年，是“十一五”规划收官之年，也是我市重大项目建设实现突破性进展、经济社会发展取得重大成就的一年。在省委、省政府和市委的正确领导下，在人大、政协的监督和支持下，我们紧紧围绕统筹推进“三化”，大力实施“三动”战略，构建“五大基地”，强化“六大建设”，推进开发区“二次创业”，全市呈现出经济强劲增长、民生得到改善、社会更加和谐的喜人局面，圆满地完成了四届人大三次会议确定的目标任务。

　　全市地区生产总值预计完成430亿元，增长19%，增速高出全省平均水平5个百分点。全口径财政收入完成32.8亿元，首次突破30亿元大关，增长41.4%；地方级财政收入完成18亿元，增长4.6%，增速均列全省第一位。规模以上工业增加值预计完成85亿元，增长33%，增速列全省第一位，高出全省平均水平14个百分点。粮食产量达到67.8亿斤，增长34.9%，创历史新高。固定资产投资预计增长41.8%，高出全省平均水平10.9个百分点。城镇居民人均可支配收入15904元，农民人均纯收入4250元，分别增长6%和20%。

　　（一）突出培育后发优势，全力推进“五大基地”建设。能源基地快速崛起。白城发电公司2×66万千瓦火电机组投产运营，白城热电厂2×20万千瓦扩建项目顺利推进。向阳、到保、来福等10个风电场启动建设，新增装机65万千瓦，全市风电总装机达到213万千瓦。新引进世界500强、全球风机制造第二的西班牙歌美飒，国内第一、世界第三的华锐集团，风电叶片国内居行业前三甲的中材科技等6户风电设备制造企业。全市油气当量达到170万吨。太阳能、生物质能等开发利用加快推进，能源产业呈现出多元化发展的新格局。粮食生产基地成效彰显。引嫩入白主体工程基本完工，大安灌区部分投入运行，镇赉、大安项目区土地开发整理81万亩。洮儿河灌区配套工程加快建设，月亮泡汉书闸工程建成使用，新开河治理项目纳入《国家中小河流治理建设规划》，全市兴水整地增粮迈上了新台阶。特色农产品加工基地不断壮大。益海嘉里、龙丹乳业、吉林金塔、东佳谷物等超亿元项目相继投产，建设了亚洲第一、世界第二的飞鹤原生态牧场等一批大型生产基地，龙头带动、基地支撑的产业化格局初步形成。区域商品集散地加快构建。欧亚购物中心一期工程建成运营。海明路步行街改造及地下人防工程开工建设。大型农产品批发市场发展到18户，区域商贸中心构架逐渐形成。全市社会消费品零售总额实现164亿元，增长20.6%。服务业增加值增长18.5%。生态产业基地扎实推进。“举市三年大造林”开局良好，完成造林40万亩，占三年造林任务40%。利用脱硫废渣改造盐碱地1800亩。生态旅游产业加快发展，安白新能源景观带等项目启动建设，大安嫩江湾被评为国家级湿地公园。全年接待国内外游客157万人次，实现收入15亿元，分别增长25%和27%。

　　（二）突出重大项目建设，大力实施“三动”战略。全市3000万元以上项目滚动发展到628个，完成投资410亿元，其中超亿元项目347个。全年开工建设项目231个，竣工投产项目55个。争取国家和省项目资金18亿元，增长53.8%。引进资金345亿元，增长36.9%。申报国家和省级科技发展计划项目37个，纳入国家创新基金、富民强县项目6个。珲乌线白城至长春高速公路提前一年竣工通车，结束了我市没有高速公路的历史。贯穿4个县（市、区）的嫩丹高速公路白城段奠基开工。白城至阿尔山铁路扩能改造启动建设，白城至长春快速铁路通过国家审批，白城长安机场进入最后审批阶段。2010年，是我市历史上投资最大、成效最好的一年，事关长远发展、多年谋划争取的重大基础设施建设项目取得了突破性进展。

　　（三）突出开发区“二次创业”，统筹推进“三化”进程。启动实施部分经济区、行政区调整改革，经济开发区扩区、工业园区建设配套服务区、洮北区接管民营经济区新建工业集中区。出台了扩权强区、封闭管理、环境整治等7个加快开发区建设的配套文件，有效地推进了“二次创业”步伐。各县（市）工业集中区建设也取得新进展，为“三化”深度融合搭建了平台。努力加快工业化进程。开工建设投资3000万元以上工业项目164个，完成投资226.6亿元，增长53.1%。实施“五个一批”改革攻坚，对通业集团等市直17户企业分类施策、盘活存量取得显著成果。特别是省市关注、停产三年的“白纺”，成功引进浙江绍兴三家知名企业，成立吉林四季盛宝纺织有限公司，完成了招商重组。去年9月份启动生产以来，实现销售收入4000万元。全市规模以上工业企业达到333户，新增69户。大力推进城镇化建设。完成了市、县新一轮城市总体规划修编工作，聘请高层次专家编制《白城新城区规划》、《白城市风貌特色规划》、《市区环城河景观带规划》、《园林绿地系统规划》等专项规划。扎实开展了“城市建设管理年”和园林城、卫生城“双城创建”活动。

　　投资13.56亿元，全面完成了市区基础设施建设十大工程、47个项目建设。开工建设占地5万平方米的阳光休闲广场，新建6条、维修8条街路。各县（市）城区、重点乡镇和新农村建设步伐较大、成效明显。着力推进农业现代化。投资5.6亿元新建各类农业园区35个。启动建设洮儿河农场标准化水田等示范区。加快推进安广农产品、福顺辣椒、东屏烤烟等5个产地专业加工园区建设。扶持培育安大牧业、嫩江湾米业等一批龙头企业，省级以上龙头企业发展到45户。裕丰集团“好雨”牌大米，被评为“中国驰名商标”。

　　（四）突出改善民生，维护社会和谐稳定。民生民利计划较好完成。城镇新增就业4.75万人，公益性岗位1.3万人，登记失业率3.85%，“零就业”家庭保持动态为零。城镇职工最低工资、企业退休人员基本养老金标准和城乡低保、新型农村合作医疗补助金额得到提高。养老、失业、工伤、生育、城镇职工和居民基本医疗保险均实现年度计划。超额完成省下达的“暖房子”工程建设任务，铺设供热管网80.3公里，撤并小锅炉37座，既有建筑节能改造50.3万平方米，获省一等奖。改造城市棚户区和危旧房175万平方米，统建廉租房14.25万平方米；改造农村泥草房5.9万户、林场棚户区1215户。又有4.2万农村人口脱贫，7.27万人口用上安全水。农村等级公路通村率达到93%。市区污水处理厂建成运行，结束了污水直接排放的历史。社会事业发展统筹推进。投资1.03亿元建设农村标准化学校15所，新建、改造中小学校舍13.4万平方米，整合农村小学校142所。完成白城中心医院、中医院新建选址和开工建设准备。公立医院改革试点加快推进，“平安医院”创建活动深入开展。手足口病防控、15岁以下人群乙肝疫苗补种、麻疹疫苗强化免疫等工作成效明显。援藏、援川、援疆卫生医疗任务较好完成。新建乡镇综合文化站19个、“农家书屋”500个、农村文化大院151个，有10个文化大院被评为“全省文化大院示范点”。以向海为实景拍摄的电视剧《永远的田野》在央视一套黄金段播出。吉剧《白沙滩》被列为全省重点精品剧目之一。白城金鹿国际电影城投入使用。双拥创建活动深入开展，部队演习支前保障、拥军忧属工作以及国防后备力量建设得到加强。全面落实人口计划生育政策，人口自然增长率控制在4‰以内。较好地完成了集体林权制度改革、国有粮食购销企业产权制度改革和电影公司转企改制等工作。“平安白城”创建活动成效显著。严厉打击违法犯罪行为，社会治安形势进一步好转，在全省群众安全感调查中我市名列前茅。高度重视信访工作，坚持主动接访下访，开展“集体访积案化解年”活动，全市信访总量下降17%。“五五”普法任务全面完成。安全生产形势总体稳定，事故发生率、死亡人数，同比分别下降5.54%和37.7%。积极做好稳定物价工作，“菜篮子”工程扎实推进。加强市场秩序、产品技术质量和食品药品安全监管，保持了侵害消费者权益重大事件和重大食品安全事故“双无”目标。强化应急管理体系建设，应对突发事件处置能力有了明显增强。

　　（五）突出打造阳光政务，加强服务型政府建设。主动接受人大的工作监督、法律监督和政协的民主监督，办理人大代表建议34件、政协委员提案69件，办结率均达到100%，满意率分别达到97%和100%。启动实施市长“直通车”、市政府领导接访约访下访、政务信息公开、人民群众监督、公开征集民意、落实反馈“六项制度”。对405项行政审批项目进行了再清理，减少行政审批项目203项。实施“12345”服务工程，百户企业评议部门软环境活动成效明显。政府绩效评估扎实推进，人事编制管理工作有效加强。加强反腐倡廉建设，坚持标本兼治，创新“制度加科技”监管方法，政府性投资、专项资金管理机制初步形成。强化从严治政，加大侵害农民利益、高考考风考纪、医疗服务等重点领域的纠风治理力度，提高了政府的公信力、执行力。

　　各位代表：

　　过去的一年，是千钧在肩、矛盾交错、艰难爬坡的一年，也是凝心聚力、众志成城、奋进跨越的一年。面对繁重艰巨的工作任务和多年积累的矛盾、困难与挑战，我们能够取得这样的成绩来之不易。这是市委总揽全局、正确领导的结果，是人大、政协有效监督、全力支持的结果，是全市各族人民戮力同心、奋勇拼搏的结果。在这里，我代表白城市人民政府，向全市各族人民，各民主党派、工商联、人民团体、无党派人士、离退休老同志，向驻白的中省直部门和企事业单位、人民解放军指战员、武警部队官兵、公安司法干警，向关心和支持白城发展的各界朋友，致以崇高的敬意和衷心的感谢！

　　同时，我们也清醒地认识到，目前我市还面临着许多亟待解决的问题，主要表现在：扩充经济总量能力弱，增长内生动力不足；农业基础差，现代设施农业发展缓慢；工业底子薄，支柱优势产业支撑不强；城市化进程步伐不快，城市建设管理滞后；民生改善欠账较多，城乡居民收入水平较低；社会管理创新不够，不稳定因素仍然存在；政府工作效能尚需进一步提高，部分工作人员服务意识差，思想保守，行为懒惰，工作缺乏主动性、创造性。对这些问题，我们要敢于面对，切实加以研究解决。

　　二、2011年工作安排

　　2011年，是实施“十二五”规划的开局之年，也是白城处在关键节点上提速跨越的重要一年。世界经济有望继续恢复性增长，但全面复苏将是缓慢艰难的过程。我国经济正处在发展与调整并重的重要战略机遇期，但国内市场竞争加剧，淘汰落后产能步伐加快，一些行业发展压力加大；货币政策由“宽松”转向“稳健”，信贷投放趋紧，融资难度加大。总体看经济发展外部环境，机遇与挑战并存，机遇蕴于挑战，机遇大于挑战。因此，我们要顺势而为，乘势而上，抢抓机遇，力争加速发展、加快转型。今年政府工作的总体要求是：深入贯彻落实党的十七届五中全会、省经济工作会议和市委四届七次全会精神，牢牢把握科学发展主题和转变方式主线，坚持“三化”统筹、“三动”并举，全力开展“五大基地攻坚年”、“二次创业竞赛年”活动，抓投资促开发、抓调整促转变、抓民生促和谐，努力实现“十二五”良好开局。

　　主要预期目标是：全市地区生产总值完成520亿元，增长18.6%。全口径财政收入超过40亿元，增长22%以上；地方级财政收入21.8亿元，增长21%。固定资产投资增长40%。社会消费品零售总额完成195亿元，增长19%。城镇居民人均可支配收入18000元，增长13.2%；农民人均纯收入4800元，增长12.9%。城镇登记失业率控制在4.6%以内。人口自然增长率控制在4‰以内。单位地区生产总值能耗下降3.2%。

　　全力做好以下七个方面工作：

　　（一）围绕“五大基地攻坚年”，全力抓项目增投资促转变

　　坚定不移实施投资拉动、项目带动，深度谋划重大项目，大力开展招商引资，切实增强经济发展后劲。

　　依托项目开发促进基地建设。围绕发展新能源产业，在推进白城发电公司二期火电项目的同时，抓紧推进大唐集团煤化工项目，协调加大石油、天然气勘探开发力度，油气开发当量达到186万吨。加快千万千瓦级风电开发，力争风电总装机超过300万千瓦。抓好通榆、洮南、镇赉“金太阳”示范工程和大安风光互补项目建设。围绕壮大特色农产品加工业，重点发展玉米深加工、燕麦开发、水稻加工等特色产业。围绕提升优势产业，抓好汽车零部件、装备制造、冶金建材、纺织服装、医药等产业发展，加大企业战略重组力度，做到靠大壮大、附强图强。围绕开发建设生态产业，发展绿色经济、低碳经济、循环经济，积极争投资、上项目，走出吉林西部特色生态产业发展路子。

　　全力以赴抓好重大项目建设。集中力量实施620个投资3000万元以上重大项目建设，完成投资500亿元。其中，亿元以上项目350个。着力推进通榆“三一”风电产业园二期、镇赉海外药业等122个续建项目建设；加快推进洮北双百新能源、正隆齿轮汽配等135个项目开工建设；积极推进环保汽车刹车片等363个项目前期工作。重点推进大安节能抽油机改造等60个以上项目竣工投产，力争洮南热电联产等20个投资亿元以上项目当年投产达效。完善重大项目建设管理机制，建立信息服务管理平台，形成项目建设的指挥、责任和监督系统。构建项目审批绿色通道，实行全程跟踪制、全程代办制，提高重大项目组织推进效能。

　　抓好招大商引大资上大项目。坚持以“长三角”、“珠三角”、“环渤海”等地区为重点，盯住央企、突出民企、注重外企，抓项目、促合作、引资金。组织开展京津鲁、沪浙苏、闽粤台等省市经贸活动，推介项目、展示白城。全市引进域外资金433亿元，增长32%。大力培育扶持大宗出口商品、出口骨干企业和外贸出口基地，扩大进出口总量，全市进出口总额达到9375万美元，增长13%。

　　（二）开展“二次创业竞赛年”活动，促进开发区跨越发展

　　强力推进开发区建设步伐，切实把开发区打造成开发开放的窗口、招商引资的阵地、项目集聚的平台、经济发展的引擎。

　　创造加快发展速度。围绕“二次创业竞赛年”活动，各开发区要坚持“发展至上、项目至重、客商至尊”，掀起招大客商、上大项目、建大企业、兴大产业的高潮。全市各开发区GDP、固定资产投资、工业增加值、全口径财政收入增幅都要达到30%以上，不断扩充经济总量、增强经济实力。

　　强化基础设施建设。经济开发区要抓好新区交通路网“二横四纵”规划，推进“七通一平”基础设施建设。工业园区要对综合配套服务区进行规划布局，引入创投基金分期开发建设，启动三期主干路网建设。查干浩特旅游经济开发区，要围绕加强基础设施建设，搞好旅游项目开发。新建洮北工业集中区要加快起步区、发展区、储备区规划和建设。加快县（市）工业集中区建设，从今年开始，力争三年内完成提档升级工作，增强县域经济发展活力。

　　着力优化服务环境。全面落实加快开发区建设的7个配套文件，深入实施“12345”服务工程。建立“一把手”责任制，完善包保服务机制，对外商投资、项目建设、企业发展实行“保姆式”服务，打造诚信白城，切实把我市建设成为外商投资的洼地、政府服务的高地、生活居住的福地。

　　（三）加快工业经济发展壮大，推进“兴工强市”进程

　　努力扩大增量、盘活存量，壮大工业经济规模。全市规模以上工业增加值达到105亿元，增长23%。

　　扶持企业做大做强。出台支持企业发展方式转变、促进工业经济快速增长的若干政策。重点扶持益海嘉里、中材科技、德尔福派克、金升镍业等10户高成长性企业。发展壮大民营经济，筛选100户成长性好的小企业，通过信贷支持等措施培育壮大，全年新增规模以上企业30户。组织实施百项重点科技项目攻关、百个新产品开发研发工程。抓好60户企业技术改造，全年完成技改投资135.6亿元，增长20%；实现新产品产值19亿元，增长18.7%。争取省级以上科技发展项目20个。

　　推进产业集群发展。研究制定全市产业集群发展规划。重点推进以华锐风机、中材叶片、国电通力为引领的风电装备产业园，以四季盛宝为中心的纺织服饰产业园，以国药集团为依托的医药产业园，以德尔福派克、金事达电气、中一精锻为龙头的汽配产业园，以中国建材集团为核心的新材料产业园，以白城发电公司为主体的煤电产业园，以金升镍业为示范的高载能产业园，以福佳机械为基础的装备制造产业园，以益海嘉里为代表的农产品加工产业园等，尽快形成一批有品牌、有规模、有影响力的产业集群。

　　破解资金瓶颈制约。开展“金融服务年”活动，完善企业信用担保体系，提高财政对中小企业担保公司的支持能力。鼓励自然人、企业法人和其他社会组织，组建担保公司和小额贷款公司。加强村镇银行建设，引进域外银行入驻我市。加强吉林敖东洮南药业、银诺克药业等7户企业上市培育，力争完成吉林金塔集团上市，实现上市公司零的突破。

　　（四）大力发展现代特色农业，加快新农村建设步伐

　　坚持以粮食增产、农业增效和农民增收为目标，促进农业发展方式转变，农业总产值实现210亿元，增长15%。

　　加快实施粮食增产工程。继续推进水利基础设施建设，完成大安灌区主体工程，加强引嫩入白配套工程建设。推进土地开发整理，完成大安项目区一期工程，启动建设大安和镇赉项目区二期工程。加快月亮??业设施工程，加强200万亩玉米膜下滴灌项目建设，实现粮食生产新跨越。

　　抓好现代农业园区发展。全市建设农业园区35个。新建、扩建100栋以上大棚瓜菜种植园区20个，建设农产品专业加工贸易园区2个。加快镇赉、大安省级生态农业示范区建设，推进洮北高效生态农业和裕丰10万亩水稻种植园区建设。加大以牧兴农、以牧富民力度，新建、改建各类牧业小区188个，建设35万吨奶牛产业园区，强化动物疫病防控和质量安全监管。抓好“菜篮子”工程，加强蔬菜生产基地建设，蔬菜种植面积达到80万亩，增强蔬菜自给能力，稳定蔬菜价格。搞好现代烟草农业试点，加快建设国家级烟叶示范区。

　　推进农业产业化经营。重点发展水稻、绿豆、葵花、蓖麻、辣椒、奶牛和肉牛、肉羊、鸭鹅兔、品牌鱼、燕麦等特色农业产业，建设“一村一品”专业乡镇5个、专业村30个。实施农产品加工龙头企业培育计划、农民专业合作组织创新计划，开工建设3000万元以上农产品加工项目10个。加快推进东佳米糠油等20个续建项目建设。扶持天福食品等一批龙头企业做大做强，省级以上龙头企业发展到50户。加强地理标志产品、特色农产品品牌培育，形成龙头带基地、基地带农户的产业化格局。

　　加快造林绿化步伐。继续深入开展“举市三年大造林”活动，推进城乡绿化美化、荒山荒地湿地治理等五大工程建设，完成造林35万亩。建设枸杞、欧李、沙枣等10个荒漠化治理示范项目基地，构建绿色生态屏障。

　　提升新农村建设水平。深入开展“样板村”创建活动，突出抓好水、电、路、气、房等基础设施建设。建成“村村通”等级公路800公里，改造农村电网1500公里，绿化村屯847个，新建村级信息服务站320个。按照村容整洁、房屋亮丽、环境优美、设施齐全的标准，建设“样板村”15个，加快新农村建设步伐。

　　（五）加速构建区域商品集散地，促进现代服务业发展

　　着重培育龙头企业，推动服务业向集群、特色、现代方向转变，加快打造连接三省（区）商贸物流中心。全年第三产业增加值实现203亿元，增长18%。

　　建设现代服务业集聚区。围绕增强城市服务功能，建设现代物流、购物消费、小商品交易、商务会展等7个服务业集聚区。重点加快建设海明路步行街地下人防工程，力争启动建设欧亚购物中心二期工程，推进白城内陆港物流中心、香港恒丰集团城市综合体、山东华达集团商贸城等项目建设，积极抓好杂粮杂豆期货交易市场前期工作，促进现代服务业提速升级。

　　推进城乡市场体系建设。规划建设黑水西瓜、蔬菜水果等特色农副产品交易集散中心。推进“万村千乡市场工程”和“新网工程”建设，建成110户标准化农家店。继续开展家电、农机、汽车、摩托车下乡以及家电“以旧换新”。大力发展资产评估、咨询服务等中介机构和金融、保险、信息等新兴服务业。

　　加强旅游资源整合开发。完善全市旅游业发展总体规划，整合向海、莫莫格、嫩江湾、查干浩特等旅游资源，打造“草原风光、湿地风貌、民族风情、风电景观”旅游品牌，推介旅游精品线路。大力推进嫩江旅游带、风电景观园等10个重点项目建设，尽快壮大生态旅游产业规模。全年接待国内外游客192万人次，实现收入19亿元，分别增长22%和25%。

　　（六）加大基础设施建设力度，加速城镇化发展步伐

　　坚持高起点规划、高标准建设、高效能管理，统筹推进城镇化，努力建设宜居宜业、绿色低碳的魅力城市。

　　突出市区建设。按照“东扩、南拓、西延”的城市总体发展方向，实施总投资34.2亿元的市区道路、环城景观带、景观大路、供排水管网、东湖湿地公园、净化亮化美化、垃圾处理等10大工程、90个项目建设。新建改造瑞光、新兴、棉纺和二环等11条街路，建设新华西路、向阳街、幸福南街3条景观大路。规划建设环城景观带，启动新城核心区5平方公里“七通一平”基础设施建设。加快26条街路、18个小区绿化改造，新增绿化面积6.3万平方米。改造供水管网4000米，完成50公里污水管网改造前期工作。实施东湖湿地公园环境治理一期工程。完成临街建筑、大型户外广告、门牌字匾等亮化美化，实施生活垃圾和医疗废弃物无害化处理工程。加快“数字白城”建设，继续抓好“城市建设管理年”，深入开展省级园林城、卫生城“双城创建”活动。加强节能减排和环境保护，推进低碳城市规划建设，争取列入国家低碳试点城市。

　　强化城镇建设。加快发展县城和小城镇，四个县（市）要立足资源禀赋、区位条件，加大县城基础设施建设力度，突出宜居、生态、安全、畅通、便利和特色，提升城市发展的质量和品位，打造具有独特风格和魅力的特色县城。加强节点镇建设，重点抓好岭下、坦途、万宝、瞻榆、安广等区位优势明显、产业特色鲜明、辐射带动突出的中心镇建设。

　　加快通道建设。加速高速公路、铁路、机场项目建设，全面推进嫩丹高速公路白城段建设进度。开工建设白城至长春快速铁路，加快白城至阿尔山铁路扩能改造。规划建设金辉公铁立交桥等3个互通出入口。全力抓好白城长安机场立项审批，协调解决建设资金问题，力争下半年开工建设。逐步构建起综合性、立体化交通大格局。

　　（七）进一步改善民生，努力让城乡居民生活得更加美好

　　围绕推进富民进程，制定实施民生民利行动计划，集中力量、集中财力解决关系人民群众切身利益的问题，使发展成果更多更好地惠及广大百姓。

　　全力保障民生民利。千方百计扩大就业，从扶持就业向鼓励创业转变，全年新增城镇就业3.8万人，公益性岗位规模达到1.4万人，确保城镇“零就业”家庭动态为零。启动就业技能提升计划，免费培训5万人。转移农村劳动力35万人。进一步增强社会保障能力，企业退休人员基本养老金标准提高13.5%。抓好镇赉、大安新型农村社会养老保险试点。推进失地农民参加养老保险工作。加快厂办大集体职工养老保险参保续保。开展“五七家属工”参加养老保险。城镇居民医保报销比例逐渐提高。工伤、生育、城镇职工基本医疗保险人数稳步增加。新型农村合作医疗参合率稳定在省定标准。城市低保月保障标准提高12.9%，农村低保年保障标准提高13.9%。实现城乡医疗救助全覆盖，符合条件的困难群众医疗救助率达到100%。失业保险金按省规定提高标准。

　　统筹发展社会事业。优化教育资源配置，抓好通榆国家义务教育均衡发展试点。积极探索职业教育“政、企、校”联盟办学模式。开展学前教育，加快乡镇中心幼儿园建设步伐。新建15所农村标准化学校。推进镇赉公立医院改革试点，加强3个县级医院、2个中心乡镇卫生院、4个城市社区卫生服务中心和20个村卫生室建设。强化文化资源共享工程示范基地建设。加快文化产业发展，做大做强风雷公司。争创中国楹联文化城市。广泛开展群众性精神文明创建活动。推进白城丹顶鹤电视剧制作中心、向海影视基地建设前期工作。加强国防教育，推进后备力量建设，完善动员工作体系。抓好双拥共建，力争实现全国“双拥模范城”三连冠。加强老龄工作，实施社区为老服务工程。坚持做好人口和计划生育工作，继续稳定低生育水平。大力推进民族宗教等工作。

　　努力办好民生实事。一是投资41.01亿元，改造城市棚户区210万平方米，建设廉租房19.7万平方米。二是投资13亿元，加快“暖房子”工程建设，新建改造热力站3座，新增供热能力375万平方米，撤并小锅炉24座，改造供热管网80公里，既有建筑节能改造50万平方米。三是争取吉林油田每日20万立方米天然气，分期分批实施“气化白城”工程，使居民用上洁净环保的天然气。四是投资2.47亿元，按三级医院标准新建建筑面积7万平方米的白城中心医院和4万平方米的白城中医院。五是投资1.3亿元，建成占地5万平方米的阳光休闲广场和建筑面积1.5万平方米的全民健身中心。六是投资3.5亿元，改造海明路步行街，建设地下人防工程。七是投资3000万元，扩建改造3700平方米的市殡仪馆。八是投资55亿元，完成农村泥草房改造98922户，林场棚户区改造1591户。九是投资3120万元，解决4万农村人口饮水安全问题。十是实施贫困村整村推进计划，实现3万农村贫困人口脱贫。

　　切实维护社会稳定。加强应急管理，强化基层应急队伍建设，有效化解各类矛盾，妥善处置突发事件。做好信访工作，严格落实领导包案、事涉部门息访和工作人员信访责任制，有效解决群众信访问题。坚持依法治市，扎实开展“六五”普法宣传。深入开展“平安白城”创建活动，严厉打击各种违法犯罪行为，构建现代化、社会化、专业化的治安防控网络。突出安全生产基础建设，毫不松懈地排查整改安全隐患，坚决遏制重特大安全事故发生。加强价格监测监管，做好稳定物价保障民生工作。继续开展食品药品安全专项整治行动，让人民群众吃得放心、用得安心。

　　三、关于《白城市国民经济和社会发展第十二个五年规划纲要（草案）》的说明

　　从2009年7月开始，市政府就成立“十二五”规划编制领导小组，聘请专家和组织相关部门开展专题研究，通过反复征求意见、修改完善，《白城市国民经济和社会发展第十二个五年规划纲要（草案）》（以下简称《纲要（草案）》），现提请大会审议。下面，我从三个方面作简要说明。

　　（一）关于“十一五”时期我市国民经济和社会发展的主要情况

　　过去五年，是白城发展进程中很不平凡的五年，是继往开来、与时俱进的五年。

　　五年来，综合经济实力显著增强。到2010年末，全市地区生产总值完成“十一五”计划的107.5%，年均增长23.1%。全口径财政收入完成“十一五”计划的156.2%，年均增长18.7%。人民生活水平明显提高。城镇居民人均可支配收入、农民人均纯收入，分别年均增长13.5%和11%。经济结构进一步优化。到2010年末，全市三次产业结构调整为19∶44∶37。生态环境不断改善。森林覆盖率提高到11.6%。基础设施建设取得重大成就。重点实施了引嫩入白、大安灌区、西部土地开发整理、珲乌线白城至长春高速公路、白城至阿尔山铁路扩能改造等重点基础设施工程。科技、教育、文化、体育、卫生等社会事业加快发展。

　　（二）关于“十二五”规划的指导思想和发展目标

　　1．指导思想。根据市委四届七次全会精神，以及我市经济社会发展的客观要求，《纲要（草案）》提出“十二五”期间全市经济社会发展的指导思想是：坚持以邓小平理论和“三个代表”重要思想为指导，以科学发展为主题，以转变经济发展方式为主线，以解放思想、更新观念为先导，以改革开放为动力，坚定发展信心不动摇，促进工业化、城镇化和农业现代化统筹发展及深度融合，实施投资拉动、项目带动和创新驱动战略，加快开发区（园区）“二次创业”步伐，建设以“五大基地”为主体的吉林西部特色经济区，全面提升地区综合实力，努力推进全市经济平稳较快发展和社会和谐进步，为全面实现小康社会奋斗目标奠定坚实基础。这一指导思想，既充分体现了省委、省政府精神，又完全符合白城实际。

　　2．基本原则。《纲要（草案）》确定了八个基本原则：一是继续保持投资稳定增长；二是进一步调整产业结构；三是坚持贯彻扩大内需战略；四是深入贯彻节约资源和保护环境基本国策；五是促进城乡区域协调发展；六是深入实施科教兴市和人才强市战略；七是改进和加强社会建设；八是加快改革开放步伐。这八个基本原则，反映了全面落实科学发展观、加快经济发展方式转变的基本要求，是推动我市经济社会转入加快发展、绿色发展、集聚发展、融入发展、借力发展、和谐发展轨道的重要保证。

　　3．发展目标。在综合考虑未来发展环境和条件的基础上，围绕经济崛起步伐加快、“三化”水平全面提升、“五大基地”形成规模、“二次创业”筑强平台、人民生活幸福美好“五大目标”，《纲要（草案）》确定“十二五”时期全市经济社会发展目标。具体包括经济总量、经济结构调整、资源利用和环境保护、人民生活水平四个方面目标。在经济总量上，要实现“三个一”目标，即到2015年，地区生产总值达到1000亿元，固定资产投资达到1000亿元，全口径财政收入达到100亿元。这些目标提出，既与白城崛起振兴任务相结合，又与全面建设小康社会目标相衔接；既强调保持较快增长速度，更注重提高发展质量和水平；既注重加快经济发展，也高度重视社会建设和民生改善。

　　（三）关于“十二五”时期的主要任务

　　根据“十二五”时期全市经济社会发展的指导思想和目标原则，《纲要（草案）》提出了经济建设、社会发展、民生改善、改革开放等10个方面主要任务。一是现代农业发展与社会主义新农村建设。农业现代化是工业化、城镇化的基础和保障。我市农业优势突出，具有发展特色现代农业的潜力和条件。要加强粮食生产基地、特色农业发展及现代农业园区建设，发展畜牧业等。二是工业经济发展及工业化建设。推动白城崛起振兴，关键要振兴工业。虽然近几年我市工业发展速度较快，但工业总量不大、结构不优、产业融合不够等问题仍然突出。要加强能源基地、特色农产品加工基地建设，以及发展汽车零部件、装备制造、冶金建材、纺织服装、医药、新兴化工等产业。三是服务业发展。加快发展服务业，是调整经济结构的重要举措。我市服务业发展总体滞后，是发展中的“短板”。要加强区域商品集散地建设，以及大力发展旅游业、金融业、信息咨询业、社区服务业和文化产业。四是基础设施建设。基础设施，是经济社会发展的基础和先导。要在抓好农业基础设施、城市基础设施等建设的同时，重点抓好铁路、公路、民航“三大通道”建设。五是城镇化建设。加快推进城镇化，是解决城乡二元结构问题、促进城乡一体化发展的必由之路。要加强城镇体系建设，加快中心城市、县级城市和重点小城镇建设。六是环境保护及生态产业基地建设。一方面，我市经济欠发达，在加快发展的同时，面临着进一步节能减排的艰巨任务。另一方面，我市生态环境脆弱，使我们更加清醒地认识到，发展生态产业、构建吉林西部生态屏障的重要性。因此，要突出抓好环境保护和生态产业基地建设。七是科学技术发展。我市具有一定的科研优势，但科研与经济结合不够紧密。要强化创新驱动和市场驱动，努力把科研优势转化为产业优势和经济优势。八是开发开放领域建设。加快白城发展必须借力发展。要加强区域交流与合作，发展对外贸易，加强招商引资和开发区（园区）建设。九是社会事业发展与建设。发展社会事业、维护社会稳定，是贯彻落实科学发展观的重要任务。要抓好教育、卫生、人口与计划生育、文化体育、广播电影电视、人民生活及社会保障事业发展，加强人才队伍建设和社会管理。十是进一步深化改革。改革是加快白城崛起振兴的现实选择。要深化行政管理体制改革，推进农村综合改革，搞好医药卫生体制改革，加快文化体制改革，切实抓好投融资体制改革等，通过改革进一步释放加快发展的新活力，增添崛起振兴的新动力。

　　各位代表：

　　全面完成今年和“十二五”期间的各项任务目标，对政府工作提出了新的更高的要求。我们必须把握发展新形势，顺应人民新期待，高举“发展”和“民生”两面旗帜，以奋勇开拓的精神状态、只争朝夕的拼搏精神、求真务实的工作作风，扎扎实实做好每一件事情。

　　要百折不挠抓发展。深入贯彻落实科学发展观，一心一意谋发展，凝心聚力抓发展，千方百计促发展，不惧风险，不畏艰难，不计得失，勇于破解矛盾和问题，在困难中大干、在大干中赶超、在赶超中实现预期目标，绝不辜负人民的期望和重托。

　　要解放思想重创新。强化政府管理，完善和改进政府及部门之间沟通协作方式，形成高效率、快节奏的行政运行机制。严格压缩一般预算支出，做到公用经费“零增长”。严格控制各类编制和机构，进一步降低行政成本。继续开展行政审批项目清理，实施行政审批流程优化提速再造。全面推行政府绩效评估，围绕全市重点工作，逐项进行责任分解，做到责任有主体、实施有监督、目标有考核，实行严格行政问责。

　　要规范行为强法治。围绕建设法治政府，切实把依法行政贯穿政府工作全过程，落实行政执法责任制。加强制度建设，用制度管权、管事、管人，确保权力规范运行，突出解决不依法办事、不执行政策、推诿扯皮甚至刁难勒索等问题。加强规范性文件监督管理，畅通行政复议渠道。

　　要提升效能转政风。全面推行阳光政务“六项制度”，以实际成效取信于民。开展软环境和政行风评议活动，进一步优化经济发展软环境。加强反腐倡廉建设，突出重点领域和关键环节治理，注重从源头上防治腐败，突出事前、事中监督，加强政府性投资和专项资金的监督管理，努力建设务实、高效、廉洁政府。

　　要接受监督促改进。不断完善监督机制，主动接受人大的工作监督、法律监督和政协的民主监督，认真办理人大代表建议和政协提案。强化监察、审计等专门监督。高度重视人民群众监督和新闻舆论监督，加强与公众直接沟通互动，以客观、透明、真实的政务信息引导公众舆论。

　　各位代表：

　　白城“十二五”发展蓝图已经绘就，我们站在新的历史起点上，处在加快发展、实现赶超的关键时期，任务十分艰巨，使命十分光荣，前途充满希望。我们要在省委、省政府和市委的正确领导下，凝聚全市人民的智慧和力量，坚定信心，团结奋斗，扎实苦干，攻坚克难，为加快白城崛起振兴、实现强市富民的目标而不懈奋斗！
